# Development and validation of a high-throughput whole cell assay to investigate *Staphylococcus aureus* adhesion to host ligands

**DOI:** 10.1074/jbc.RA120.015360

**Published:** 2021-01-13

**Authors:** Laurenne E. Petrie, Allison C. Leonard, Julia Murphy, Georgina Cox

**Affiliations:** College of Biological Sciences, Department of Molecular and Cellular Biology, University of Guelph, Guelph, Ontario, Canada

**Keywords:** high-throughput screening, anti-adhesives, antivirulence, Staphylococcus aureus, MRSA, antibiotic resistance, cell wall-anchored proteins, methicillin-resistant Staphylococcus aureus (MRSA), bacterial adhesion, virulence factor, drug discovery

## Abstract

*Staphylococcus aureus* adhesion to the host's skin and mucosae enables asymptomatic colonization and the establishment of infection. This process is facilitated by cell wall-anchored adhesins that bind to host ligands. Therapeutics targeting this process could provide significant clinical benefits; however, the development of anti-adhesives requires an in-depth knowledge of adhesion-associated factors and an assay amenable to high-throughput applications. Here, we describe the development of a sensitive and robust whole cell assay to enable the large-scale profiling of *S. aureus* adhesion to host ligands. To validate the assay, and to gain insight into cellular factors contributing to adhesion, we profiled a sequence-defined *S. aureus* transposon mutant library, identifying mutants with attenuated adhesion to human-derived fibronectin, keratin, and fibrinogen. Our screening approach was validated by the identification of known adhesion-related proteins, such as the housekeeping sortase responsible for covalently linking adhesins to the cell wall. In addition, we also identified genetic loci that could represent undescribed anti-adhesive targets. To compare and contrast the genetic requirements of adhesion to each host ligand, we generated a *S. aureus* Genetic Adhesion Network, which identified a core gene set involved in adhesion to all three host ligands, and unique genetic signatures. In summary, this assay will enable high-throughput chemical screens to identify anti-adhesives and our findings provide insight into the target space of such an approach.

An important component of bacterial virulence is adhesion to the host's skin and mucosae (1, 2, 3, 4); as such, the development of anti-adhesive therapeutics could provide significant clinical benefits (1, 3, 5). However, such an approach requires an in-depth knowledge of adhesion-associated factors in clinically relevant strains (6). In addition, sensitive and robust assays are required to enable high-throughput chemical screening campaigns.

This study is focused on the opportunistic pathogen *Staphylococcus aureus.* The organism's arsenal of virulence factors enables a variety of diseases ranging from skin and soft tissue infections to severe systemic infections (7). Strains of *S. aureus* possess numerous cell wall-anchored (CWA) surface proteins (8) covalently attached to the peptidoglycan through the action of sortases, mainly the housekeeping transpeptidase sortase A (SrtA) (9, 10). CWA proteins perform many functions, including adhesion, immune evasion, iron acquisition, and biofilm formation (8). As such, the pathogenicity of SrtA-deficient *S. aureus* strains is significantly impaired in several animal models of infection (11, 12, 13). In regard to adhesion, a subset of CWA proteins recognize different host ligands, which enables *S. aureus* strains to colonize and cause infections in distinct sites within the body (8). Therefore, specific anti-adhesives will be required in different clinical contexts.

One bottleneck in the study of *S. aureus* adhesion is the lack of a high-throughput and whole cell assay. Rather than focusing on a single target (*e.g.* SrtA), a whole cell assay would permit an unbiased phenotypic approach for identifying anti-adhesives, enabling the sampling of a larger target space. Furthermore, whole cell assays provide inhibitors with guaranteed biological activity, which is often a limiting factor of target-based approaches (14, 15). Existing adhesion detection methods have largely relied on staining of adhered bacteria with crystal violet, or the manual enumeration of colony-forming units (16, 17, 18, 19, 20). Although these assays are capable of detecting strains with reduced adhesion, they lack the feasibility, consistency, sensitivity, and/or speed required for high-throughput applications. To overcome these limitations, we describe the development of an ELISA to detect *S. aureus* adhesion to host ligands in a high-throughput manner.

To validate our high-throughput screening assay, and to gain insight into the genetic requirements of *S. aureus* host cell adhesion, we performed a large-scale genetic screen, profiling an arrayed transposon mutant library of methicillin-resistant *S. aureus* (MRSA) USA300 (21). Specifically, we investigated the genetic requirements of *S. aureus* adhesion to three different host cell ligands: fibronectin, keratin, and fibrinogen. These ligands are implicated in distinct and clinically relevant scenarios, as detailed below.

### Fibronectin

*S. aureus* can invade, survive, and replicate within professional and non-professional phagocytic cells (22, 23). The interaction of *S. aureus* with fibronectin underlies the organism's ability to invade non-professional phagocytic host cells, which is mediated by the fibronectin-binding proteins (FnBPs) A and B (24, 25, 26, 27, 28, 29). Host cell invasion facilitates the establishment of chronic and difficult to treat infections (24, 30, 31, 32); the intracellular lifestyle of *S. aureus* protects the bacterium from antibiotics (33) and the host's immune system (34). Furthermore, endothelial host cell invasion can cause inflammation, endocarditis, and crossover into blood vessels, resulting in systemic infections (35). Interfering with *S. aureus* fibronectin adhesion could provide significant clinical impact; in this instance, anti-adhesives would likely act as adjuvants (36) potentiating antibiotics in infected individuals and could reduce the occurrence of chronic infections.

### Keratin

The ability of *S. aureus* to colonize the skin and nasal cavity is facilitated by the CWA adhesin clumping factor B (ClfB), which recognizes analogous binding motifs within keratin and cornified envelope proteins such as loricrin, located within the epidermis (1, 37, 38, 39, 40, 41). *S. aureus* persistently colonizes the nasal cavity of ∼20% of the adult population (42, 43) and the majority (∼80%) of aggressive hospital infections are caused by these colonizing isolates (1, 44, 45, 46). Interfering with ClfB-mediated adhesion to the skin could enable the development of *S. aureus* decolonizing agents (1).

### Fibrinogen

Finally, the interaction of *S. aureus* with fibrinogen/fibrin is important during the early stages of infection (47). *S. aureus* adhesion to fibrinogen is associated with the colonization of prosthetic joints and/or indwelling devices (48), cell clumping, and the formation of a protective fibrin/fibrinogen shield; the latter two of which are important for virulence and immune evasion (49, 50). Although *S. aureus* produces an array of factors interacting with fibrinogen, clumping factor A (ClfA) is the adhesin enabling the abovementioned pathogenic traits (51). Consequently, ClfA has been the focus of *S. aureus* vaccine efforts (52).

Although the molecular basis and importance of the abovementioned *S. aureus* host-ligand interactions are well-described, it is likely that numerous cellular factors contribute to adhesion, which are all candidate anti-adhesive targets. Indeed, virulence gene expression is controlled by intricate networks of regulators (53) and the surface stability of adhesins is governed by the controlled production of extracellular proteases, which degrade adhesins and/or host ligands (54, 55, 56, 57, 58). As such, our high-throughput genetic screen identified 20 adhesion defective mutants and we used this information to construct a *S. aureus* Genetic Adhesion Network. Overall, we identified a core gene set involved in adhesion to all three host ligands and we delineated unique genetic signatures. Although the genetic screen validated our high-throughput whole cell assay, identifying a significant proportion of know adhesion-associated factors, we also identified previously undescribed genetic loci that warrant further investigation. These new loci may represent undescribed drug targets and this assay is well suited to identify new small molecule inhibitors of all the targets identified in this study.

In summary, this whole cell assay will enable strategies to inhibit *S. aureus* host cell adhesion, providing a means to profile large chemical libraries, and also sheds light on the target space of such an approach. The identification of anti-adhesive therapeutics could provide an important complement to traditional antibacterial chemotherapy, for use as single or combinatorial therapies, potentially reducing the need for antibiotics and/or enhancing antibiotic action (36) during infection.

## Results

### The development of a whole cell high-throughput assay to detect surface adhered S. aureus

**Figure 1:**
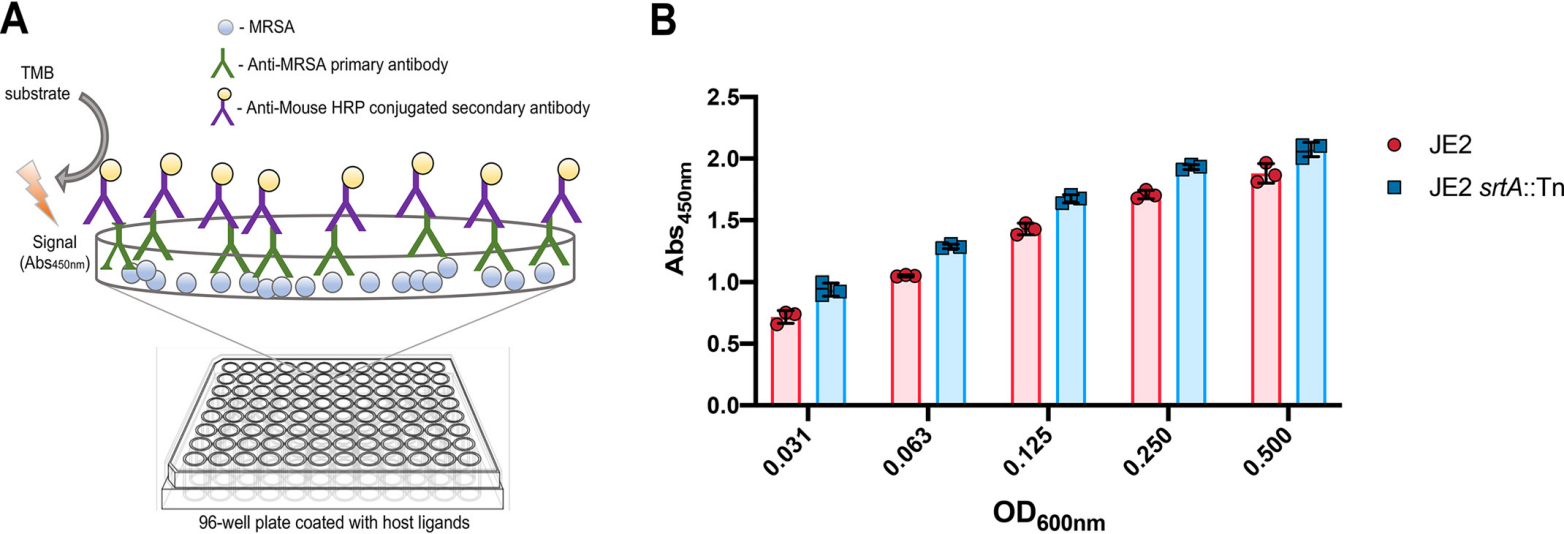
**An ELISA-based approach for the large-scale profiling of *Staphylococcus aureus* adhesion to host ligands.***A,* schematic diagram of the high-throughput *S. aureus* adhesion assay. *B,* recognition of surface-adhered MRSA USA300 by the primary antibody is not dependent on the presence of cell wall-anchored proteins. A Nunc™ MaxiSorp™ microtiter plate was coated with MRSA USA300 JE2 and isogenic JE2 *srtA*::Tn (devoid of CWAs) propagated to an OD_600nm_ of 0.6. The cells were harvested, washed, and standardized to the various optical densities shown. Using the described ELISA, detection of surface adhered JE2 *srtA*::Tn was equivalent to that of the parental strain, indicating that recognition by the primary antibody is independent of CWA surface proteins. The values shown are the average mean ± S.D. of three duplicate biological replicates.

To enable the large-scale profiling of *S. aureus* adhesion to host ligands, here we describe the development of a whole cell high-throughput assay (Fig. 1*A*). We selected the predominant community-associated MRSA strain in North America: MRSA USA300 (59, 60), which is designated CMRSA-10 in Canada (59, 61, 62, 63, 64). Due to the clinical importance of this lineage, a genetic resource (the Nebraska Transposon Mutant Library: the NTML) has been developed to enable the study of gene function and regulation using the MRSA USA300 JE2 strain as a background (21). Transposon mutants from the NTML will herein be referred to by their respective gene name followed by ::Tn.

To detect surface-adhered MRSA USA300, we developed an ELISA-based approach, selecting a mouse monoclonal primary antibody with affinity for intact and UV-treated *S. aureus*, and a horseradish peroxidase-conjugated secondary antibody. To optimize the assay, we first assessed detection of MRSA USA300 JE2 adhered to the surface of MaxiSorp microtiter plates (*i.e.* nonspecific and high-affinity binding to an abiotic surface) (Fig. 1*B*). The ELISA successfully detected the surface-adhered bacteria, and we then compared detection of an isogenic *srtA*::Tn strain (21) adhered to this abiotic surface, which was equally well-recognized (Fig. 1*B*). This strain is devoid of the majority of CWA proteins (including the immunoglobulin binding protein A), revealing that the primary antibody recognizes factors other than CWA surface proteins and is, therefore, suitable to study CWA adhesin-mediated adhesion.

**Figure 2:**
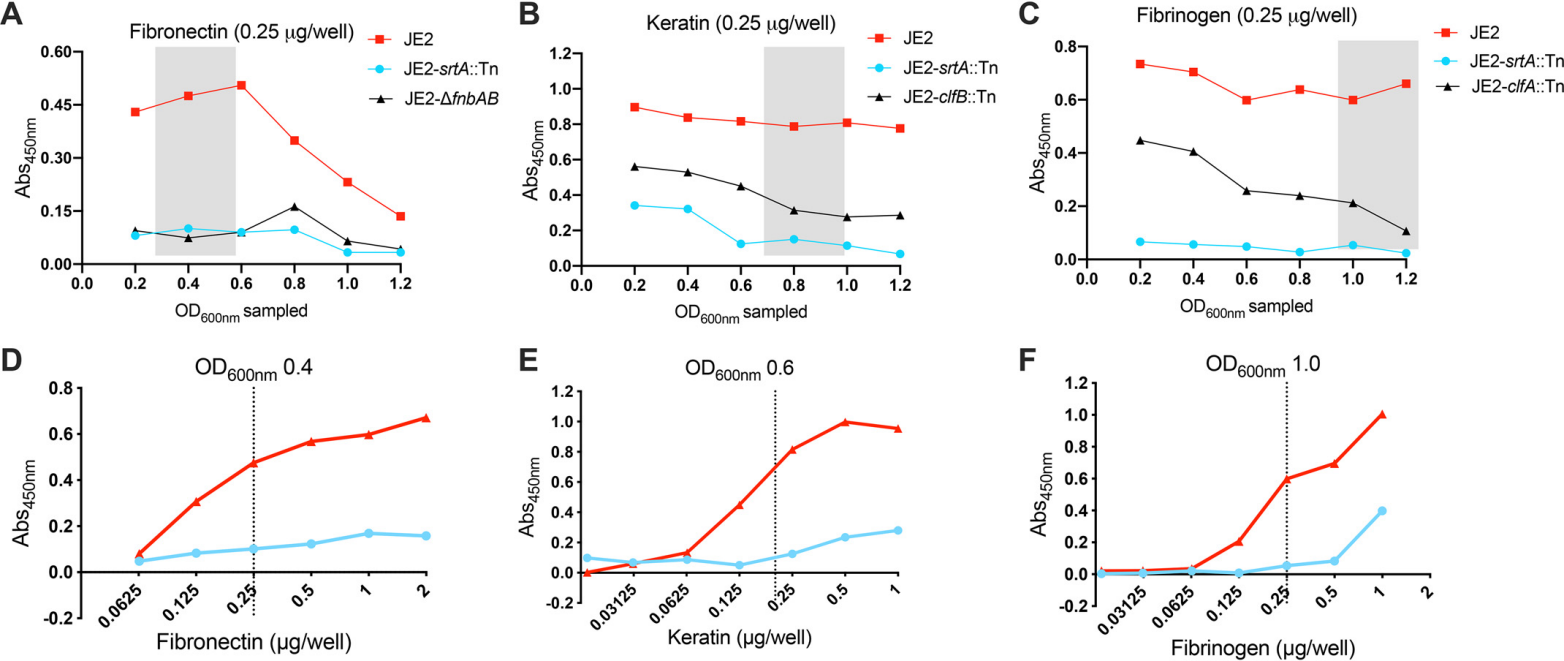
**Optimization of the whole cell adhesion assay.** The affinity of *S. aureus* for host ligands changes throughout the cell cycle; the strains were sampled at the different OD_600nm_ shown and standardized to an OD_600nm_ of 1.0. Adhesion to fibronectin (*A*), keratin (*B*), and fibrinogen (*C*), was assessed using the described ELISA (as shown in Fig. 1*A*), by measurement of the *A*_450nm_. For each host ligand, subsequent adhesion studies were performed with strains grown to the OD_600nm_ range highlighted by the *gray box*. This range was selected based on the OD_600nm_ where MRSA USA300 JE2 adhesion was maximal and presented an optimum ratio of separation between MRSA USA300 JE2 and the isogenic mutants of each respective ligand. To optimize the screening window, dose-dependent ELISAs were performed with MRSA USA300 JE2 and isogenic JE2 *srtA*::Tn adhering to human-derived fibronectin (*D*), keratin (*E*), and fibrinogen (*F*). The strains were grown to the specified OD_600nm_ highlighted by the *gray box* and standardized to an OD_600nm_ of 1.0. The *dashed lines* indicate the chosen concentration of each ligand for the subsequent transposon library adhesion screens. The values shown are the mean of three technical replicates.

We next investigated *S. aureus* adhesion to host ligands, using microtiter plates coated with either human-derived fibronectin, keratin, or fibrinogen. It is well described that the affinity of *S. aureus* for host ligands changes throughout the cell cycle (37, 65, 66, 67, 68, 69), which needs to be taken into consideration when profiling adhesion to different ligands. Thus, we harvested *S. aureus* at different stages in the cell cycle, with standardized and equivalent colony-forming units, and assessed adhesion to each host ligand (Fig. 2, *A*–*C*). To further validate the assay, we also profiled isogenic mutants devoid of the keratin adhesin ClfB (*clfB*::Tn (21)), fibronectin adhesins FnBPA/B (Δ*fnbA* and *fnbB* (70)), fibrinogen adhesin ClfA (*clfA*::Tn (21)), as well as *srtA*::Tn (21), which lacks the majority of CWA proteins (Fig. 2, *A*–*C*). As anticipated, *S. aureus* lacking CWA proteins (*srtA*::Tn) was severely impaired in adhesion to all three ligands. In line with previous studies, fibronectin adhesion was maximal during the earlier stages of growth (OD_600nm_ 0.2-0.5) (67, 68) and significantly decreased thereafter (Fig. 2*A*). CWA protein-mediated keratin adhesion (Fig. 2*B*) was maximal at an OD_600nm_ > 0.6 (37, 65, 66). Although ClfB is also capable of adhering to fibrinogen (71), we confirmed loss of the gene encoding ClfA significantly impairs fibrinogen binding later in the growth cycle (OD_600nm_ >1.0) (65, 69) (Fig. 2*C*). In addition, ClfA-mediated fibrinogen adhesion was isolated by the inclusion of CaCl_2_. The presence of Ca^2+^ is known to interfere with the interaction of both ClfA (72) and ClfB (65, 72) with fibrinogen. However, ClfB is more sensitive (65, 72) (IC_50_= 0.8 mm, *versus* 2-3 mm for ClfA) and a ClfA subinhibitory level of Ca^2+^ (1.7 mm) was selected when assessing fibrinogen adhesion. Finally, to provide a robust and sensitive screening window, we titrated each host ligand and selected a single concentration for subsequent studies (Fig. 2, *D* and *E*).

### Validation of the high-throughput whole cell S. aureus adhesion assay

**Figure 3:**
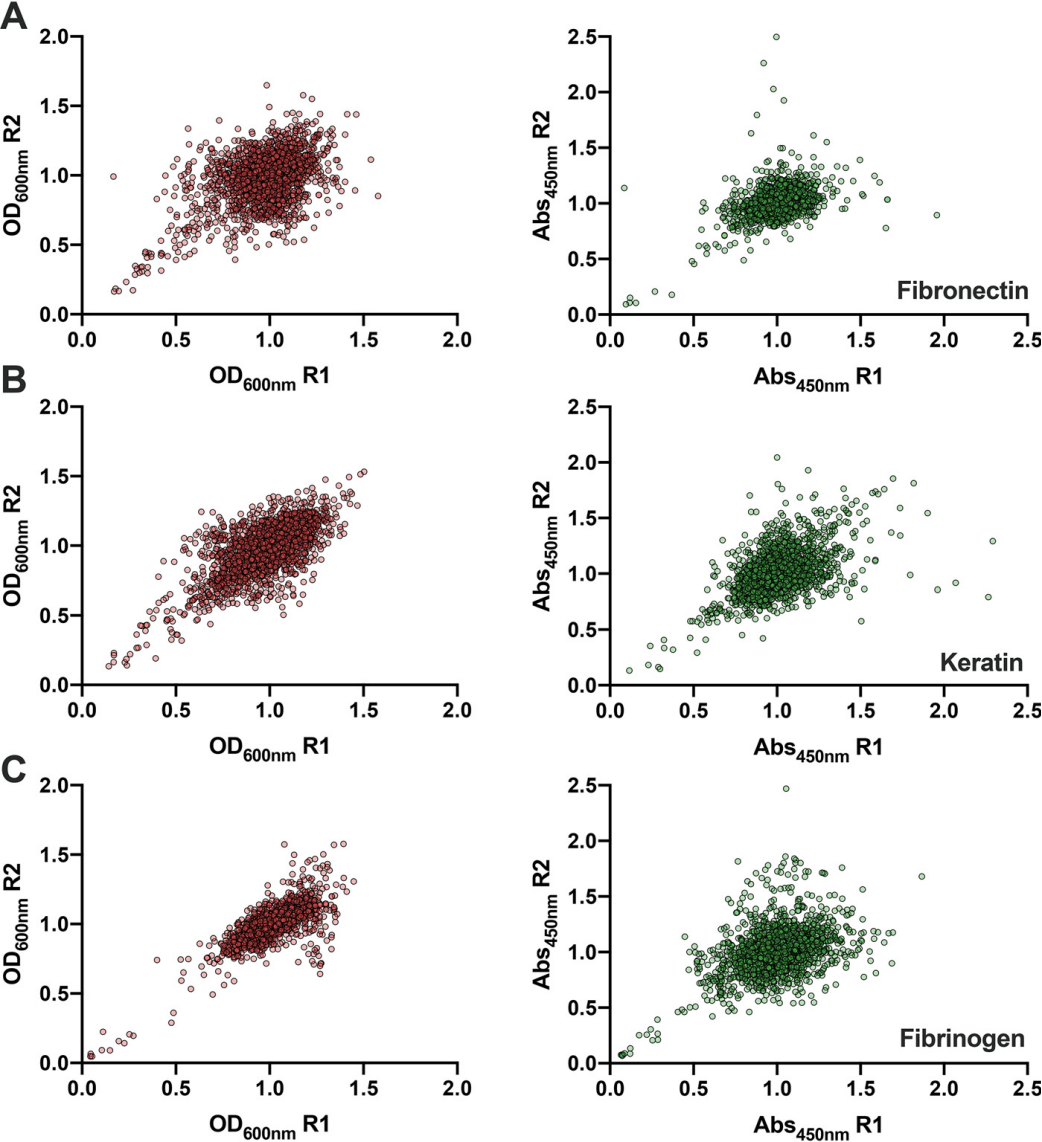
**Replica plots of normalized growth and adhesion values from the large-scale profiling of an MRSA USA300 transposon library (**21**).** Growth (OD_600nm_) is shown on the left (*red*) and adhesion (*A*_450nm_) values are shown on the right (*green*). *A,* fibronectin; *B,* keratin; *C,* fibrinogen. *R1* = replicate 1 and *R2* = replicate 2.

**Figure 4:**
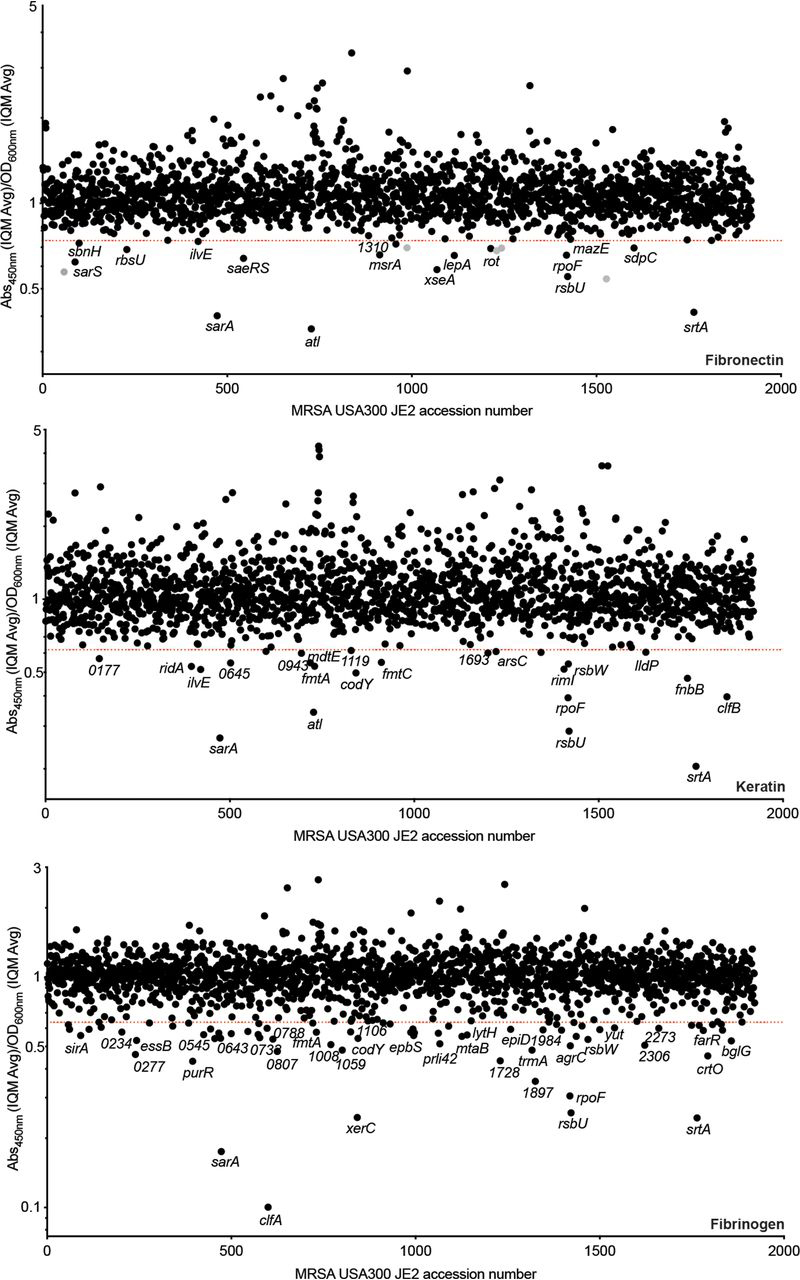
**Identifying genetic loci associated with *S. aureus* adhesion to human-derived fibronectin, keratin, and fibrinogen, respectively.** The values shown in the index plots are the ratio between the average of duplicate adhesion values (*A*_450nm_) and duplicate growth values (OD_600nm_) (see Fig. 3). The strains are ordered based on their associated SAUSA300 accession number (21). The screens were performed with ligand concentrations and strains sampled at the given time points depicted in Fig. 2. Strains falling below the *red lines* exhibited a ratio less than 4 standard deviations (fibronectin and keratin) and 6 standard deviations (fibrinogen) from the IQM (75) of the data set.

To validate the use of our assay for the large-scale profiling of *S. aureus* adhesion, we took a functional genomics approach to identify genetic loci contributing to adhesion to fibronectin, keratin, or fibrinogen. We used an MRSA USA300 transposon library comprised of 1,920 annotated mutants generated by the insertion of the mariner-based *bursa aurealis* transposon into genes that are not considered to be essential for growth under standard laboratory conditions (21). In total, this library provides coverage of ≥75% of the predicted MRSA USA300 genes (73). For profiling of the individual mutants, the library was arrayed into a 96-well–microtiter plate glycerol stock. A cryo-replicator was used to inoculate the library into tryptic soy broth (TSB) in 96-well–round bottom microtiter plates. After 18 h at 37 °C, these cultures were used to inoculate fresh TSB, and the strains were grown at 37 °C until the OD_600nm_ range shown in Fig. 2*A* was reached, which depends on the host ligand being profiled. The growth of each mutant was measured, revealing reproducible growth (Fig. 3), and enabled the identification of strains with growth defects. The bacterial cells were then applied to microtiter plates coated with the individual host ligands and the ELISA was performed, which reproducibly detected adhesion (Fig. 3). To circumvent the limitations of plate-to-plate variation, the growth and adhesion values were normalized by the interquartile mean (IQM) (74). The IQM represents the mean of the inner two quartiles of ranked data, thereby preventing any influence from outliers (74). We then determined the ratio between adhesion and growth for each mutant (Fig. 4). A hit was defined as a mutant that provided a ratio less than 4 standard deviations for keratin and fibronectin, and less than 6 standard deviations for fibrinogen, from the IQM of the data set. Because the mutants were sampled at a higher OD_600nm_ in the fibrinogen screen, the variance was lower and a higher cut-off value was used.

As anticipated, the assay was validated by the identification of known adhesion-related determinants, such as the gene encoding SrtA, which was identified in all three screens. We also identified disruptions within genes encoding key host ligand adhesins: *clfB* in the keratin screen and *clfA* in the fibrinogen screen (Fig. 4). Because there are two fibronectin binding proteins (FnBPA/B), disruption of either gene alone is not sufficient to impair fibronectin adhesion (Fig. 4).

### Construction of a S. aureus genetic adhesion network highlights the anti-adhesive target space

**Figure 5:**
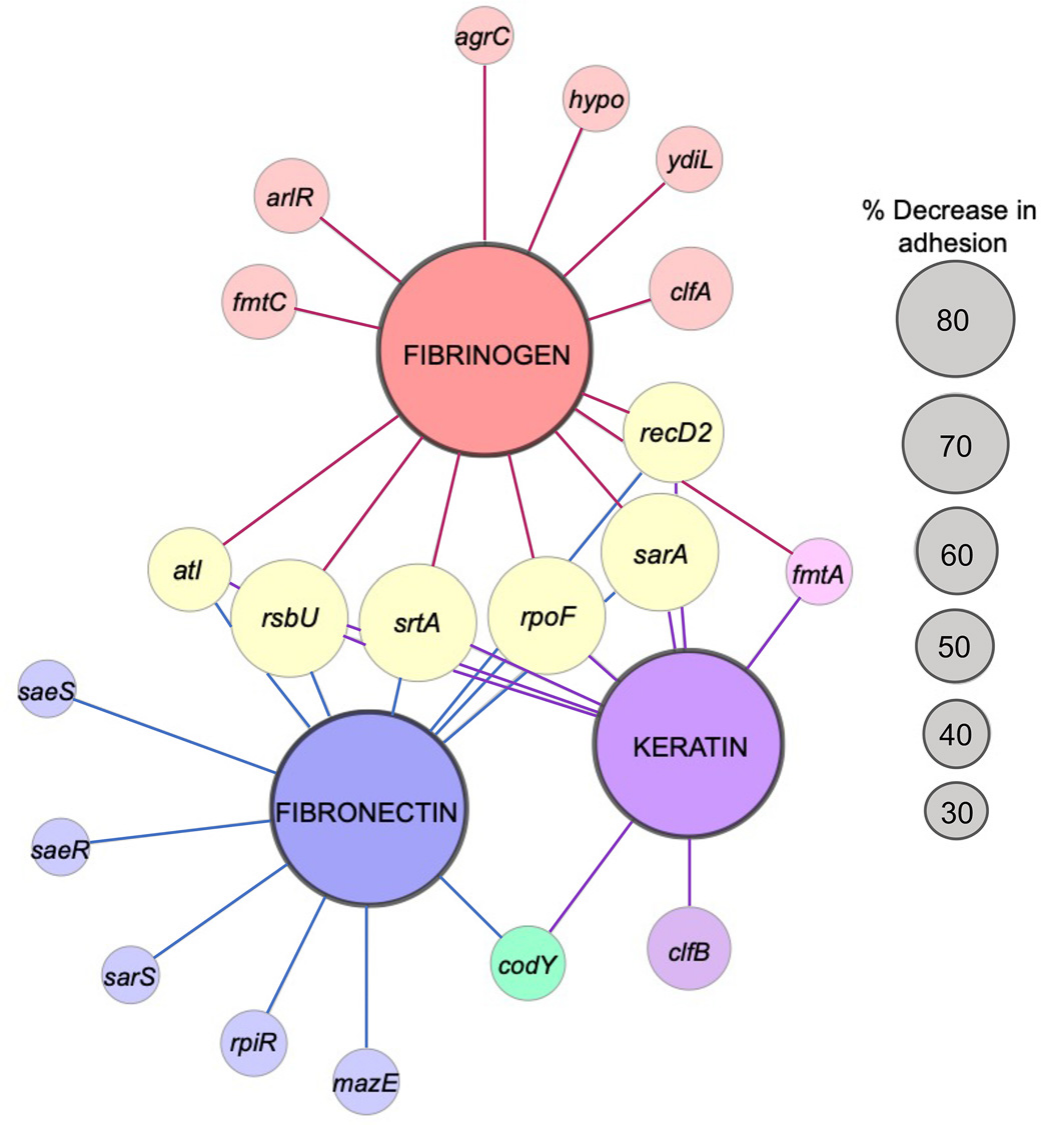
**The *S. aureus* Genetic Adhesion Network and anti-adhesive target space.** The larger central nodes identify the host ligand in each screen (fibrinogen, fibronectin, and keratin). The smaller nodes radiating from these points indicate transposons inserted into genes that caused a significant reduction (Student's *t* test, *p* value ≤ 0.05; see Table 1 and Fig. S1) in adhesion to the host ligand they are linked. The node size for each gene reflects the percent adhesion compared with the parental strain (*i.e.* the larger the node the more pronounced the attenuated adhesion). The figure was generated using Cytoscape. Genes *mazE* (SAUSA300_2026), *recD2* (SAUSA300_1576), *rpiR* (SAUSA300_2264), and *ydiL* (SAUSA300_1984) are names provided on the basis of their predicted structure and function. One gene, labeled *hypo* (hypothetical protein), is unannotated (SAUSA300_0602).

All adhesion attenuated mutants identified in the primary adhesion screens (Fig. 4) were compiled into a sublibrary and profiled for adhesion to all three host ligands, as described above. For mutants that showed confirmed adhesion-related phenotypes, the transposons were transduced using phage 80α into a fresh JE2 background, to eliminate the possibility of secondary-site mutations. Transposon insertions were confirmed by PCR and the newly generated mutants were then profiled to assess dose-dependent adhesion to each host ligand (Fig. S1). A single concentration of each ligand was selected to compare the relative adhesion of each mutant to the parental strain (Table 1). This information was used to create a visual representation of the contribution of each gene to adhesion (Fig. 5). By comparing adhesion of each strain to each of the three polymers we observed similarities and differences between the three screens. In total, we identified 20 genetic loci associated with adhesion to at least one of the three host ligands; 12 for fibronectin, 9 for keratin, and 13 for fibrinogen. We also identified a core gene set required for adhesion to all three host ligands and we identified undescribed genetic loci. Importantly, these genes represent potential targets that could be identified in future screens of chemical libraries. To further strengthen our findings, we profiled poorly characterized adhesion-defective mutants (*sarS, rpiR, fmtC, fmtA*, *recD2*, *hypo*, and *ydiL*) using crystal violet as an alternative detection method (Fig. S2). By employing this method, with the exception of *sarS* in the fibronectin screen, we confirmed that all of the mutants displayed reduced adhesion, which is not a reflection of reduced affinity of the detecting antibodies.

**Table 1:** The *S. aureus* Genetic Adhesion Network Genes associated with *S. aureus* adhesion to a minimum of one of three host ligands (keratin, fibronectin, and fibrinogen) Adhesion was assessed by titrating each host ligand (Fig. S1). For creation of the Genetic Adhesion Network, a final concentration was selected for each ligand, as indicated. The value listed for each mutant strain is the average percentage of adhesion relative to wild-type JE2 (three biological replicates), with lower values indicating a greater reduction in adhesion to a specific ligand. Genes *mazE* (SAUSA300_2026), *recD2* (SAUSA300_1576), *rpiR* (SAUSA300_2264), and *ydiL* (SAUSA300_1984) are provisional names provided on the basis of their predicted structure and function. One gene, labeled hypo, is unannotated (SAUSA300_0602). *p* values were calculated using the two-tailed unpaired Student's *t* test; statistically significant (*, *p* ≤ 0.05) decreases in adhesion, compared with wild-type JE2, were used to create the Genetic Adhesion Network (Fig. 5).

Gene	Locus	Keratin (0.125 μg/well) % adhesion	Keratin *p* value	Fibronectin (0.125 μg/well) % adhesion	Fibronectin *p* value	Fibrinogen (0.25 μg/well) % adhesion	Fibrinogen *p* value
*agrC*	SAUSA300_1991	106.8	0.57	102.4	0.75	77.0	0.040*
*arlR*	SAUSA300_1308	107.4	0.33	111.5	0.25	57.9	0.00096*
*atl*	SAUSA300_0955	51.6	0.026*	23.7	0.0015*	50.7	0.0044*
*clfA*	SAUSA300_0772	104.0	0.72	130.1	0.20	44.4	0.00050*
*clfB*	SAUSA300_2565	42.2	0.0055*	81.4	0.45	101.9	0.26
*codY*	SAUSA300_1148	68.3	0.038*	49.4	0.012*	97.2	0.80
*fmtA*	SAUSA300_0959	60.5	0.028*	73.2	0.12	76.7	0.049*
*fmtC*	SAUSA300_1255	96.1	0.44	82.7	0.13	54.5	0.00093*
*hypo*	SAUSA300_0602	100.4	0.95	100.4	0.97	65.1	0.026*
*mazE*	SAUSA300_2026	98.9	0.92	68.9	0.0058*	96.3	0.75
*recD2*	SAUSA300_1576	41.8	0.00029*	38.4	0.0019*	20.6	0.000082*
*rpiR*	SAUSA300_2264	116.6	0.050	67.1	0.0056*	136.9	0.020
*rpoF*	SAUSA300_2022	18.6	0.0013*	25.7	0.0012*	35.2	0.0018*
*rsbU*	SAUSA300_2025	16.5	0.0012*	17.0	0.00082*	35.3	0.0016*
*saeR*	SAUSA300_0691	108.1	0.43	79.6	0.030*	121.7	0.056
*saeS*	SAUSA300_0690	104.9	0.44	86.0	0.037*	117.5	0.016
*sarA*	SAUSA300_0605	26.0	0.00016*	29.6	0.000051*	17.7	0.000085*
*sarS*	SAUSA300_0114	104.3	0.55	70.4	0.014*	116.1	0.14
*srtA*	SAUSA300_2467	20.9	0.0015*	21.4	0.00083*	34.4	0.0016*
*ydiL*	SAUSA300_1984	102.8	0.86	115.7	0.068	69.0	0.015*

### Assessing the proteolytic activity of adhesion attenuated mutants

**Figure 6:**
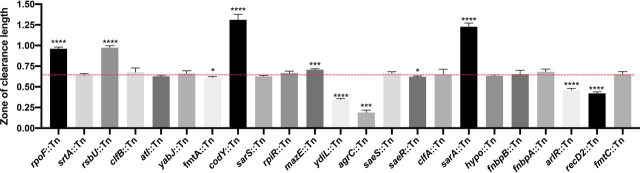
**Assessing the level of proteolytic activity in the adhesion attenuated mutants.** Protease activity was assessed using TSA containing 1.5% Difco Skim Milk. The zones of clearance were measured using ImageJ. Each strain was measured with at least three biological replicates. Welch's *t* test (*, *p* ≤ 0.05; **, *p* ≤ 0.01; ***, *p* ≤ 0.001; ****, *p* ≤ 0.0001) was used to compare the mean length for each mutant to the mean of the parental strain (*red dashed line*).

We identified a number of genes associated with virulence gene regulation, and three were identified in all three screens: *rpoF* (the alternative sigma factor B; SigB; σ^B^) (75), *rsbU* (75) (increases SigB activity; *rsbW* (75) was also identified, which is a polar mutation), and the gene encoding the global staphylococcal accessory regulator (*sarA*) (76). These genes are known to impact the expression of a wide variety of virulence-related genes, including the genes encoding adhesins (77, 78). In addition to positively regulating adhesin gene expression (76, 79), a number of the identified regulators (*e.g.* SarA and CodY*)* also repress extracellular protease production (54). Therefore, disruptions within these repressors would enhance the mutant's proteolytic capabilities. Some of these proteases are known to impact the stability of surface proteins (55, 56) and/or host ligands, enabling the bacterium to detach and disseminate (54, 57, 66). The NTML has previously been profiled to identify mutants with protease activity, which highlighted 12 mutants (*brnQ, ilvE, sarA, xerC, hslU, codY, rpoF, rsbW, rsbV, rsbU*, NE1833) with increased production, many of which had previously been associated with this phenomenon (21). As shown in Fig. 5, a number of these mutants also exhibit reduced adhesion. We assessed the level of proteolytic activity of the newly generated mutants on casein plates (Fig. 6), by measuring the zone of clearance, which enabled us to compare the extent of protease production for each mutant. Overall, we identified varying degrees of proteolysis, with five mutants exhibiting enhanced protease production and six with decreased levels (Fig. 6).

## Discussion

The antibiotic resistance crisis signifies an urgent need for orthogonal and complementary therapeutics to control and treat bacterial infections. Antivirulence approaches (80), including the inhibition of bacterial host cell adhesion (1), could reduce the severity of this global health crisis. However, the development of such strategies requires sensitive and robust assays amenable to high-throughput applications, and an in-depth understanding of the underlying processes. Here, we present an ELISA-based approach (Fig. 1) for the high-throughput profiling of *S. aureus* adhesion to host ligands. We performed a genetic screen to confirm the assay was able to detect genetic loci previously associated with adhesion, and to ascertain the MRSA USA300 anti-adhesive target space. Overall, the NTML enabled us to assess the contribution of ≥75% of predicted MRSA USA300 genes (73). During creation of the NTML, the authors acknowledged 579 open reading frames were not interrupted, a significant fraction of which are likely indispensable for growth (21). Therefore, profiling the NTML enabled assessment of a large proportion of the dispensable genes in MRSA USA300.

In this work we identified 20 genetic loci (1% of the NTML) associated with adhesion to at least one of the three clinically important host ligands. As anticipated, many of the identified genes have previously been implicated in adhesion. For example, in addition to identifying the *srtA* mutant, which is devoid of CWAs, the *clfB* mutant was identified in the keratin screen, and *clfA* in the fibrinogen screen. In addition, we also identified the gene encoding the major peptidoglycan hydrolase, autolysin (Atl), which reduced adhesion to all three host ligands (Fig. 5 and Fig. S1). Atl, and the functionally interchangeable enzyme in *Staphylococcus epidermidis* (AtlE) (81), have been described as staphylococcal adhesins (82). These proteins have been associated with adhesion to polystyrene (82) and host proteins including fibronectin (83, 84), fibrinogen (84), vitronectin (84, 85), heparin (83), gelatin (83), heat shock cognate 71-kDa protein (Hsc70) (86), and thrombospondin 1 (86). Here, we show that keratin is a host cell ligand also affected by loss of Atl.

Generation of a Genetic Adhesion Network identified a core subset of genes (*n* = 6) required for adhesion to all three ligands (Fig. 5 and Table 1), which included expected genes such as *srtA* (11), *sarA* (87, 88, 89), *rpoF* and *rpoF*-related genes (*rsbU*) (77, 78), and *atl* (83, 84). Inhibitors of these elements could represent anti-adhesives capable of simultaneously interfering with the interaction of *S. aureus* with fibronectin, keratin, and fibrinogen.

To the best of our knowledge, the adhesion-associated roles of *sarS, rpiR, fmtC, fmtA*, *recD2*, *hypo*, and *ydiL* (Fig. 5, Fig. S2, Table 1) remain unknown. Of note, we were unable to confirm the adhesion-associated defect of *sarS* using an alternative detection method (Fig. S2). In addition, genomic assessment and operon predictions (90) showed that some of these genes are located within an operon (*e.g. recD2*), and thus we cannot rule out polar effects due to transposon insertion. Complementation studies are required to further confirm the adhesion phenotypes.

Based on our current knowledge, we summarize the findings of this study by broadly categorizing each identified genetic locus (Fig. 5) into four likely and/or known groups: 1) adhesin gene expression (*saeRS*, *codY*, *rsbU*, *rpoF*, *sarA*); 2) adhesin surface presentation (*srtA, arlRS*); 3) extracellular protease production (*rpoF*, *rsbU*, *codY, saeRS, sarA*); and 4) unknown/unconfirmed roles (*sarS, rpiR, mazE (rpoF), fmtC, fmtA, recD2, hypo, ydiL*). In terms of adhesin expression (Group 1), we identified a number of genes associated with virulence gene regulation: *rpoF* (75, 77, 78), *rsbU* (75, 77, 78), and *sarA* (76, 87, 88, 89). These genes are known to impact the expression of genes encoding adhesins (77, 78). Furthermore, deletion of *codY* reduced the affinity of *S. aureus* for keratin and fibronectin (Fig. 5) and CodY has been shown to positively influence the expression of the fibronectin adhesin *fnbA* (79). In regard to adhesin surface presentation (Group 2), SrtA covalently attaches adhesins to the peptidoglycan (9, 10), and disruption of the ArlRS two-component system reduces fibrinogen adhesion due to increased production of the Giant Staphylococcal Surface Protein (encoded by the *ebh* gene), preventing fibrinogen recognition by ClfA (91). As described, in addition to positively regulating adhesin gene expression (76, 79), a number of the identified regulators (*e.g.* SarA and CodY) also repress extracellular protease production (Group 3) (54, 55, 56, 57, 66), which can degrade surface proteins and/or host ligands. Indeed, increased protease production (*e.g.* due to loss of SarA repression (55)) attenuates virulence, and complete loss of protease activity causes a hypervirulent phenotype, which could be due to the increased stability of surface-associate virulence factors (57, 58). Because we identified a number of genes that have previously been associated with protease production, we assessed the proteolytic activity of the newly created mutants (Fig. 6) (54). Overall, we identified a significant enrichment of mutants with varying degrees of protease production. Importantly, in addition to degrading adhesins, extracellular proteases can also degrade host ligands. This study sampled both possibilities simultaneously, because we applied the bacterial cells and the spent growth medium, containing secreted proteases, to immobilized ligands. Proteolytic assessment could be used to provide insight into the targets of candidate anti-adhesives identified using the described whole cell assay; for example, if the inhibitors enhance proteolysis, they could be targeting the genetic loci identified in Fig. 6. Finally, whereas the role of MazE (Group 4) in *S. aureus* adhesion is currently unknown, the *mazE* gene resides in the *mazEF/pemIK* toxin-antitoxin loci, which directly precedes the σ^B^-operon. Disruption of this gene could impact the expression of *rpoF*.

In summary, here we describe the development and validation of a sensitive and robust whole cell assay to identify anti-adhesive therapeutics. We also provide the first comprehensive functional genomic approach to ascertain the factors directly contributing to *S. aureus* host cell adhesion. Delineating the genetic requirements of *S. aureus* adhesion has provided a more in-depth understanding of the interaction of this organism with the host, and sheds light on the MRSA USA300 anti-adhesive target space. The development of anti-adhesive therapeutics would complement our antibiotic arsenal, offering the potential to reduce the severity of the antibiotic resistance crisis using a more targeted approach. As shown in Table 1 and Fig. 5, this assay is capable of detecting mutants with varying degrees of adhesion, which suggests it will be well suited for chemical library screening, enabling the identification of anti-adhesives with differing degrees of potencies.

## Materials and methods

### Bacterial strains, plasmids, and growth conditions

The NTML (NR-48501) and MRSA USA300 JE2 (21) were obtained from the Network on Antimicrobial Resistance in *S. aureus* (NARSA) repository through BEI resources, NIAID, National Institutes of Health (RRID:SCR_013698). The NTML was constructed in the MRSA USA300 JE2 strain, which was used as a background for all purposes of this study. Regeneration of the adhesion-attenuated mutants of interest was accomplished by transducing the transposons into the parental JE2 strain using phage 80α transduction. Successful transductants were selected on erythromycin (5-10 μg/ml) and confirmed by PCR using gene- and transposon-specific primers (Table 2). Unless otherwise stated, *S. aureus* strains were propagated in BD Bacto^TM^ Tryptic Soy Broth. For profiling of the NTML, the strain library was arrayed in a 96-well–microtiter plate glycerol (25% v/v) stock format at −80 °C. A cryo-replicator and replicator press (EnzyScreen) were used to inoculate 100 μl of TSB in 96-well–round bottom microtiter plates. All strains were grown at 37 °C with aeration at 220 rpm (25 mm throw), with the exception of microtiter plates that were aerated with 900 rpm using an incubator with a 3-mm throw (INFORS HT Multitron).

**Table 2:** Primers used in this study

Primers	Sequence (5′-3′)	Transposon primer used	Source
**Confirmation of *bursa aurealis* transposon insertion site**			
Upstream	CTCGATTCTATTAACAAGGG		Bae *et al*. (2008)
Buster	GCTTTTTCTAAATGTTTTTTAAGTAAATCAAGTAC		Bae *et al*. (2008)
New_Upstream	CTTCAAACTTGACTTCAGC		This study
New_Buster	CCAGTCTGGATCCAGTTG		This study
accC_Tn_check	TTCGTTGTTTAATTGCG	Buster	This study
arlR_Tn_check	TTGATTACGGTGCAGA	Upstream	This study
atl_Tn_check	GTTGCATTAACGCTTGTAG	Upstream	This study
clfA_Tn_check	AACACGCAATTCGGAA	Buster	This study
clfB_Tn_check	TTGAAAAAAAGAATTGATTATTTGTC	Buster	This study
codY_Tn_check	ACGAGAGAGTTAAACACG	Upstream	This study
fmtA_Tn_check	GGTTGCGCCGTCTAAAC	fmtA_r *versus*_check	This study
fmtA_r*versus*_check	CACCCTTCGTATTGTAAGG	fmtA_Tn_check	This study
fmtC_Tn_check	GGCATCGCTTGTTATTC	Upstream	This study
fnbA_Tn_check	CTTAGGTACGGCATTAG	Buster	This study
fnbB_Tn_check	GCAATCTTAGATACGGC	Buster	This study
GNAT4_Tn_check	GAGACTTGTTTCGACAG	Upstream	This study
graR_Tn_check	TGGGTGATATGGATGC	Upstream	This study
hypo_r*versus*_check	GCAAGTGGCAACTCTATTG	New_Buster	This study
ilvE_new_check	GCAATCACCATGTCACAAG	ilvE_r*versus*_check	This study
ilvE_r*versus*_check	GTACAACGACTCTCCAAC	ilvE_new_check	This study
mazE_Tn_check	TGATTAGACGAGGAGATG	Upstream	This study
PutLipo_Tn_check	GACACTGGGATGTTTAC	Upstream	This study
RecD2_Tn_check	TGTCAGACCCTACACT	Upstream	This study
rot_Tn_check	GCATAAGTTAGCACATACAA	Buster	This study
rpiR_Tn_check	CGCGTTAAACAACAATAGC	New_Upstream	This study
rpoF_Tn_check	GCGAAAGAGTCGAAATC	Buster	This study
rsbU_Tn_check	GCCTGAAGACATTGTCG	Upstream	This study
rsbW_r*versus*_check	TTATCGAAATGCGCG	Buster	This study
sarA_Tn_check	GAGTTGTTATCAATGGTCAC	New_Upstream	This study
sarS_Tn_check	AGTGCATATACAAGGAGA	NaT_fwd_check	This study
sdcS_NaT_Tn_check	GTGCGGGACAACTTATTG	NaT_fwd_check	This study
NaT_fwd_check	GCACTGATTAAGTTTACCC	SdcS_NaT_Tn_check	This study
spa_r*versus*_check	CTAGGTGTAGGTATTGCATC	New_Buster	This study
srtA_Tn_check	TTATTTGACTTCTGTAGCTACAAAGATTTTACG	Upstream	This study
srtB_Tn_check	TTAACTTACCTTAATTATTTTTGCGAC	Upstream	This study
vraG_Tn_check	TGGCGTTAATTATGACC	Upstream	This study
xerC_Tn_check	GAATCATATTCAAGATGCGT	Upstream	This study
yabJ_r*versus*_check	CACAACAAGATTACCGG	Upstream	This study
YdiL_Tn_check	GGGCATCATTGCTAACTG	New_Buster	This study
YfeH_Tn_check	ATGTTGCGACATTAGG	Upstream	This study

### Antibodies and reagents

Surface adhered *S. aureus* was detected using a mouse anti-*S. aureus* monoclonal primary antibody raised against UV-inactivated *S. aureus* (Invitrogen) and a secondary anti-mouse goat IgM, HRP-conjugated antibody (Invitrogen). HRP colorimetric substrate (1-step^TM^ Ultra 3,3‘,5,5‘-tetramethylbenzidine) and Nunc^TM^ Immunosorbent (Maxisorp^TM^) 96-well–microtiter plates were purchased from Thermo Scientific. Maxisorp^TM^ plates were coated with keratin derived from human epidermis and fibronectin and fibrinogen from human plasma (Millipore Sigma). BSA (BSA, HyClone^TM^) was solubilized in PBS.

### The ELISA-based S. aureus adhesion assay

For coating Maxisorp^TM^ plates with host ligands, 100 μl of keratin (diluted in carbonate buffer: 15 mm Na_2_HCO_3_, 35 mm NaHCO_3_ (pH 9.6)), fibrinogen, or fibronectin (diluted in PBS (pH 7.4)), were applied and incubated at 4 °C for 18 h. The plates were blocked with 300 μl of 2% BSA (w/v) for 1.5 h at room temperature. The microtiter plate lids were removed, and the bacterial cultures were irradiated under UV light for 20 min and stored at 4 °C. 100 μl of each culture was applied to the host ligand-coated microtiter plate wells, the plates were sealed, agitated for 5 min (900 rpm), and incubated for 1 h with gentle shaking (100 rpm). 100 μl of the primary antibody (2 μg/ml in 1% BSA) was applied to each well and the plates were agitated for 5 min (600 rpm), followed by 1 h incubation with gentle shaking (100 rpm). 100 μl of the HRP-conjugated secondary antibody (125 ng/ml in 1% BSA) was applied as described for the primary antibody. Adhesion was assessed using 100 μl of room temperature equilibrated colorimetric substrate (3,3‘,5,5‘-tetramethylbenzidine), which was agitated for 1 min (600 rpm), followed by gentle shaking (100 rpm) for 20 min. The reaction was quenched by oxidation with 100 μl of 2 m H_2_SO_4_. Absorbance was measured at 450 nm using a BioTek Synergy H1 microplate reader. Between each described step, the plates were incubated at room temperature and washed four times with 300 μl of PBS (pH 7.4), with the exception of host ligand aspiration, where the plates were only washed once with 300 μl of PBS. When assessing fibrinogen adhesion, the PBS was supplemented with 1.7 mm CaCl_2_.

To optimize the described assay, the strains were sampled at different stages of the growth cycle. Overnight saturated cultures were diluted 1:100 in TSB and the bacterial cells were harvested via centrifugation (4000 × *g*, 4 °C) at intervals of 0.2 from an OD_600nm_ of 0.2 to 1.2. The cells were washed with PBS and standardized to an OD_600nm_ of 1.0. Simultaneously, the polymer and antibodies concentrations were varied until an optimal signal to noise ratio was achieved, by assessment of the Z-factor (92). The parental MRSA USA300 JE2 strain and an isogenic *srtA*::Tn mutant strain were used as positive and negative controls, respectively.

MRSA USA300 primary antibody recognition was determined by coating a Nunc™ MaxiSorp™ microtiter plate with MRSA USA300 JE2 and isogenic JE2 *srtA*:Tn. Overnight saturated cultures were diluted 1:100 in TSB and the strains were propagated to an OD_600nm_ of 0.6. The cells were harvested via centrifugation (4000 × *g*, 4 °C), washed with PBS, and standardized in PBS to an OD_600nm_ of 0.5, 0.25, 0.125, 0.0625, and 0.0313. The standardized cultures were applied to an uncoated Nunc™ MaxiSorp™ microtiter plate and incubated for 1 h with gentle agitation (100 rpm). Following incubation, the plates were blocked, and the ELISA was performed as described.

### Crystal violet adhesion assay

Nunc™ MaxiSorp™ microtiter plates were coated with ligands, blocked, and washed as described. Overnight cultures were diluted 1:100 in 10 ml of TSB in 50-ml tubes. To assess fibrinogen and keratin adhesion, 100 μl of each culture, propagated to the respective OD_600nm_, was applied to ligand-coated microtiter plate wells, the plates were sealed, agitated for 5 min (900 rpm), and incubated for 1 h with gentle shaking (100 rpm). The bacterial cultures were irradiated under UV light for 20 min. For fibronectin adhesion, due to the lower OD_600nm_, 200 μl of each culture was applied to fibronectin-coated microtiter plate wells and the plates were treated as described, this step was repeated twice. Adhesion was assessed by staining adherent cells with 100 μl of filtered 0.5% (w/v) crystal violet for 2 min, washing three times in 300 μl of PBS (pH 7.4), and solubilizing in 0.7% (v/v) acetic acid. Absorbance was measured at 595 nm using a BioTek Synergy H1 microplate reader.

### Profiling the NTML to identify adhesion attenuated mutants

The NTML was profiled using a cryo-replicator (EnzyScreen) to inoculate 100 μl of TSB (5 μg/ml of erythromycin), followed by incubation at 37 °C for 18 h with aeration (900 rpm). These saturated cultures were then used to inoculate 100 μl of TSB and the strains were grown to the appropriate OD_600nm_ for each host ligand (Fig. 2). The adhesion assay was performed as described above. Data were ranked and normalization was performed by using the IQM method (74). Ranked data were divided into four quartiles where the mean of the inner two quartiles represents the IQM (74). Normalization per plate was achieved by comparing each strain to the IQM (74).

To confirm the adhesion attenuated phenotypes, the newly generated transposon mutants were propagated in microtiter plates as described. MaxiSorp™ plates were coated with increasing concentrations of fibronectin, keratin, or fibrinogen, starting at a high dose of 1 μg/well (10 μg/ml). The ELISA was performed as described above, with a minor modification. Following UV irradiation, 50 μl of each culture and 50 μl of PBS were applied to the host ligand-coated microtiter plate wells. MRSA USA300 JE2 was included in each plate as a control. Each strain was assessed using a minimum of three biological replicates. The resulting data were used to generate the genetic adhesion network, which was created using Cytoscape (93). GraphPad Prism 8 was used for statistical analysis.

### Assessing mutant proteolytic activity

Proteolytic activity was assessed on TSA containing 1.5% Difco Skim Milk. Cultures were inoculated into 100 μl of TSB and propagated overnight at 37 °C with aeration (900 rpm). Each strain was point inoculated onto the skim milk agar, from the overnight culture, using a cryo-replicator. Plates were incubated at 37 °C for 21 h before being imaged. Zones of clearing were assessed using ImageJ (94), by measuring from the edge of the spotted colony to the edge of the zone of clearance. Each strain was measured with at least three biological replicates. GraphPad Prism 8 was used for statistical analysis. Welch's *t* test was used to compare the mean length for each mutant to the mean of the parental strain (JE2).

## Data availability

The authors confirm that the data supporting the findings of this study are available within this article (and its supporting material). The raw data from the primary genome-wide adhesion screens are available upon request. Please contact the corresponding author (gcox@uoguelph.ca).
