# Supplementary material for: Development and validation of a high-throughput whole cell assay to investigate Staphylococcus aureus adhesion to host ligands
Source: J Biol Chem. 2021 Jan 13;295(49):16700–12. doi: 10.1074/jbc.RA120.015360 (PMC7864066; doi:10.1074/jbc.RA120.015360)
Supplement: Supplementary file 1 [file mmc1.pdf]

Supporting information: Development and validation of a high-throughput whole cell assay to investigate *Staphylococcus aureus* adhesion to host ligands

Laurenne E. Petrie[1§], Allison C. Leonard[1§], Julia Murphy[1] & Georgina Cox[1*]

[1]College of Biological Sciences, Department of Molecular and Cellular Biology, University of Guelph, 50 Stone Rd E, Guelph, Ontario, Canada N1G 2W1

[*]Corresponding author: Dr. Georgina Cox, College of Biological Sciences, Department of Molecular and Cellular Biology, University of Guelph, 50 Stone Rd E, Guelph, Ontario, Canada N1G 2W1. Tel: +1 519-824-4120, Email: gcox@uoguelph.ca

[§]These authors contributed equally

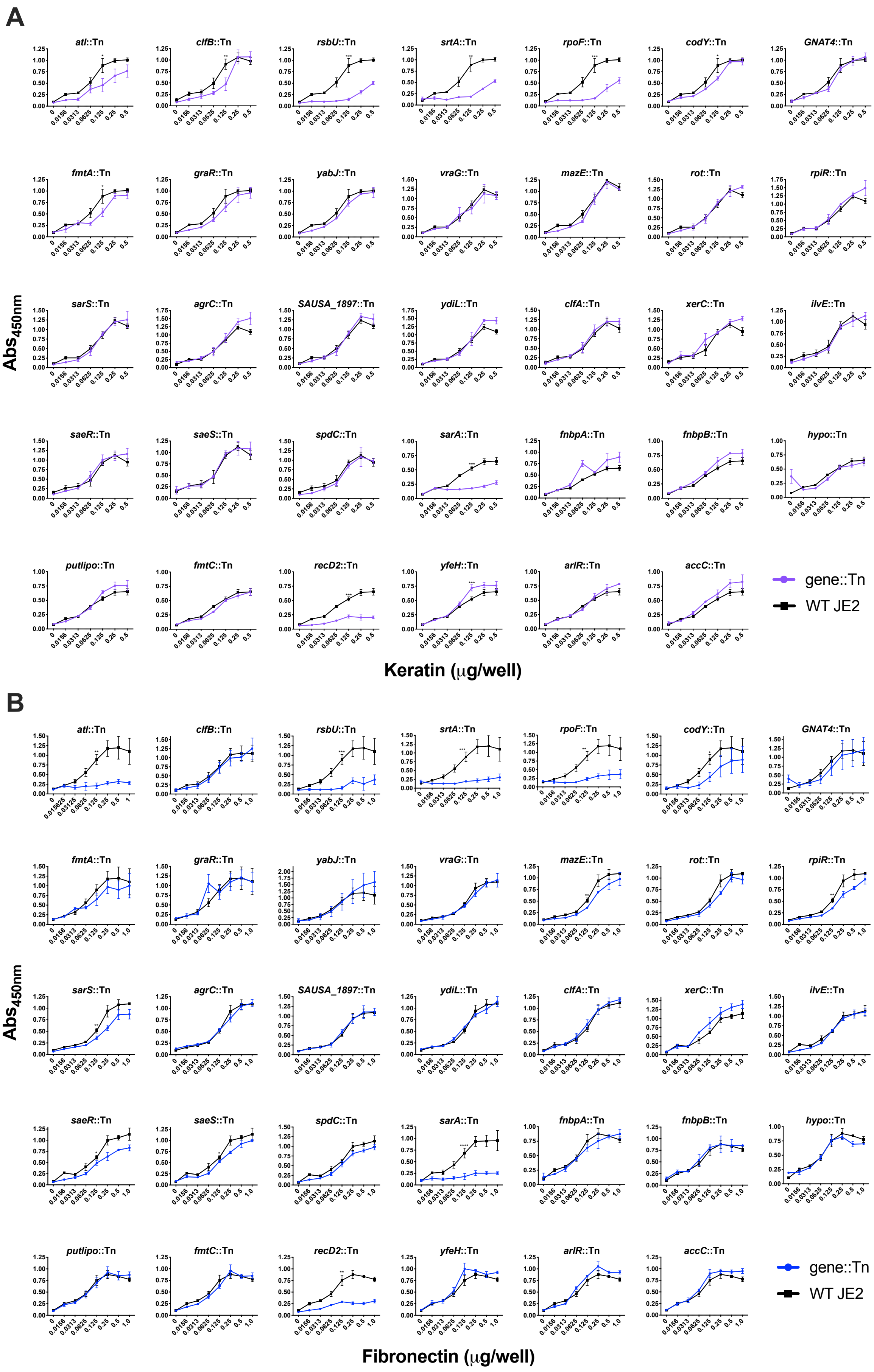
A
atl::Tn
clfB::Tn
rsbU::Tn
srtA::Tn
rpoF::Tn
codY::Tn
GNAT4::Tn
fmtA::Tn
graR::Tn
yabJ::Tn
vraG::Tn
mazE::Tn
rot::Tn
rpiR::Tn
sarS::Tn
agrC::Tn
SAUSA_1897::Tn
ydiL::Tn
clfA::Tn
xerC::Tn
ilvE::Tn
saeR::Tn
saeS::Tn
spdC::Tn
sarA::Tn
fnbpA::Tn
fnbpB::Tn
hypo::Tn
putlipo::Tn
fmtC::Tn
recD2::Tn
yfeH::Tn
arlR::Tn
accC::Tn
gene::Tn
WT JE2
Abs450nm
Keratin (μg/well)
B
atl::Tn
clfB::Tn
rsbU::Tn
srtA::Tn
rpoF::Tn
codY::Tn
GNAT4::Tn
fmtA::Tn
graR::Tn
yabJ::Tn
vraG::Tn
mazE::Tn
rot::Tn
rpiR::Tn
sarS::Tn
agrC::Tn
SAUSA_1897::Tn
ydiL::Tn
clfA::Tn
xerC::Tn
ilvE::Tn
saeR::Tn
saeS::Tn
spdC::Tn
sarA::Tn
fnbpA::Tn
fnbpB::Tn
hypo::Tn
putlipo::Tn
fmtC::Tn
recD2::Tn
yfeH::Tn
arlR::Tn
accC::Tn
gene::Tn
WT JE2
Abs450nm
Fibronectin (μg/well)

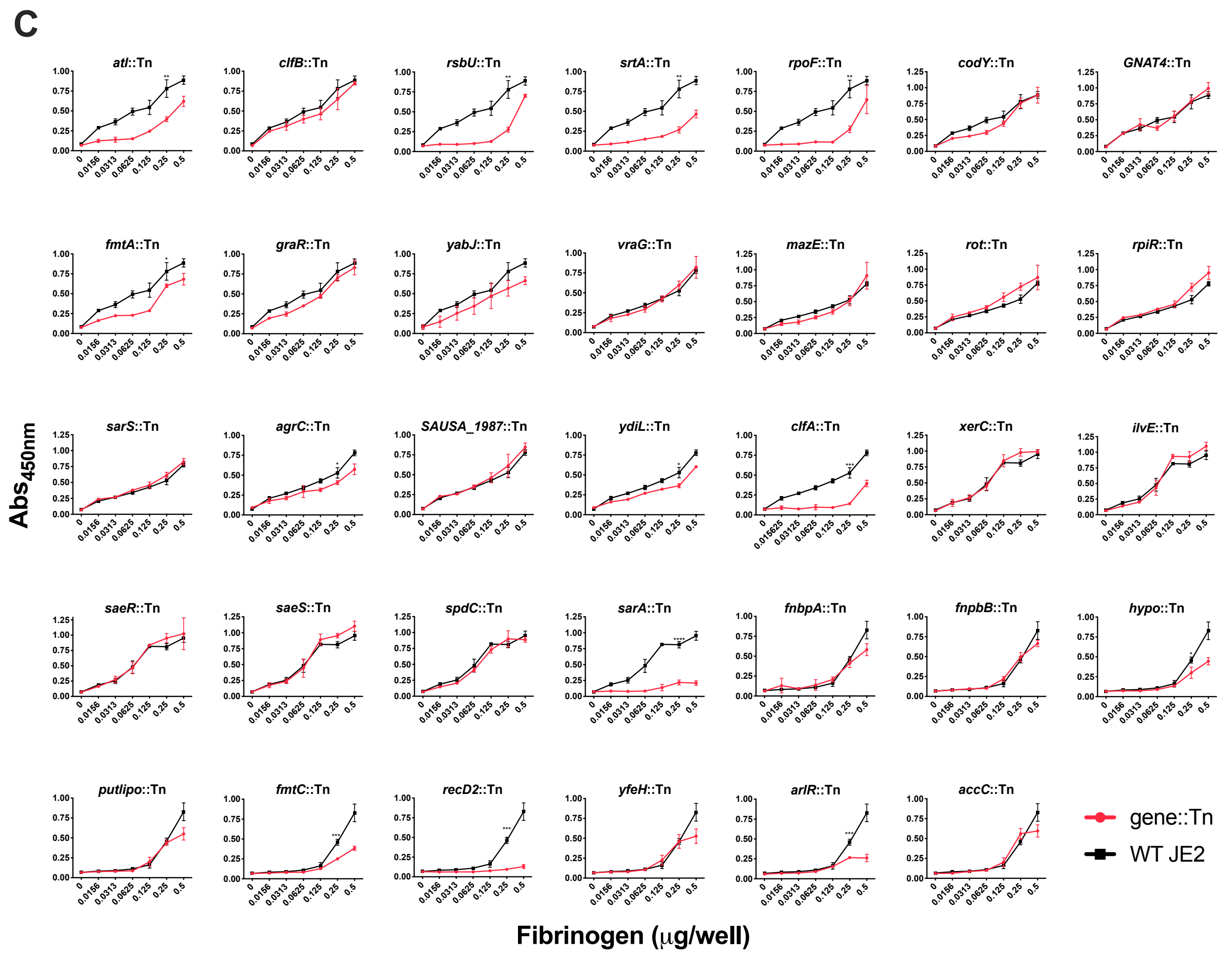

Figure S1. Construction of a *S. aureus* Genetic Adhesion Network: genes associated with *S. aureus* adhesion to a minimum of one of three host ligands (A. Keratin, B. Fibronectin, and C. Fibrinogen). Adhesion attenuated mutants identified in Figure 4 were compiled into a sub-library and profiled for adhesion to all three host ligands. For mutants that showed confirmed adhesion-related phenotypes, the transposons were transduced using phage 80α into a fresh JE2 background, to eliminate the possibility of secondary-site mutations. These newly generated mutants (n=34 shown above) were profiled to confirm reduced adhesion. Each ligand was titrated (A-C), and the mutant strains (shown in each individual graph) were propagated in microtiter plates to the appropriate OD_{600nm}, with three biological replicates. The ELISA was performed to assess adhesion (Abs_{450nm}) and the values shown are the average of three biological replicates ± the standard deviation. A final concentration was selected for each ligand (keratin 0.125 ug/well; fibronectin 0.125 ug/well; fibrinogen 0.25 ug/well) and was used to calculate the average percentage of adhesion relative to wild-type JE2 propagated in the same microtiter plate (see Table 1). *P*-values were calculated using the two-tailed unpaired Student's *t*-test; statistically significant ($P \leq 0.05$*; $P \leq 0.01$**; $P \leq 0.001$***; $P \leq 0.0001$****) decreases in adhesion, compared to wild-type JE2, were used to create the Genetic Adhesion Network (Figure 5).

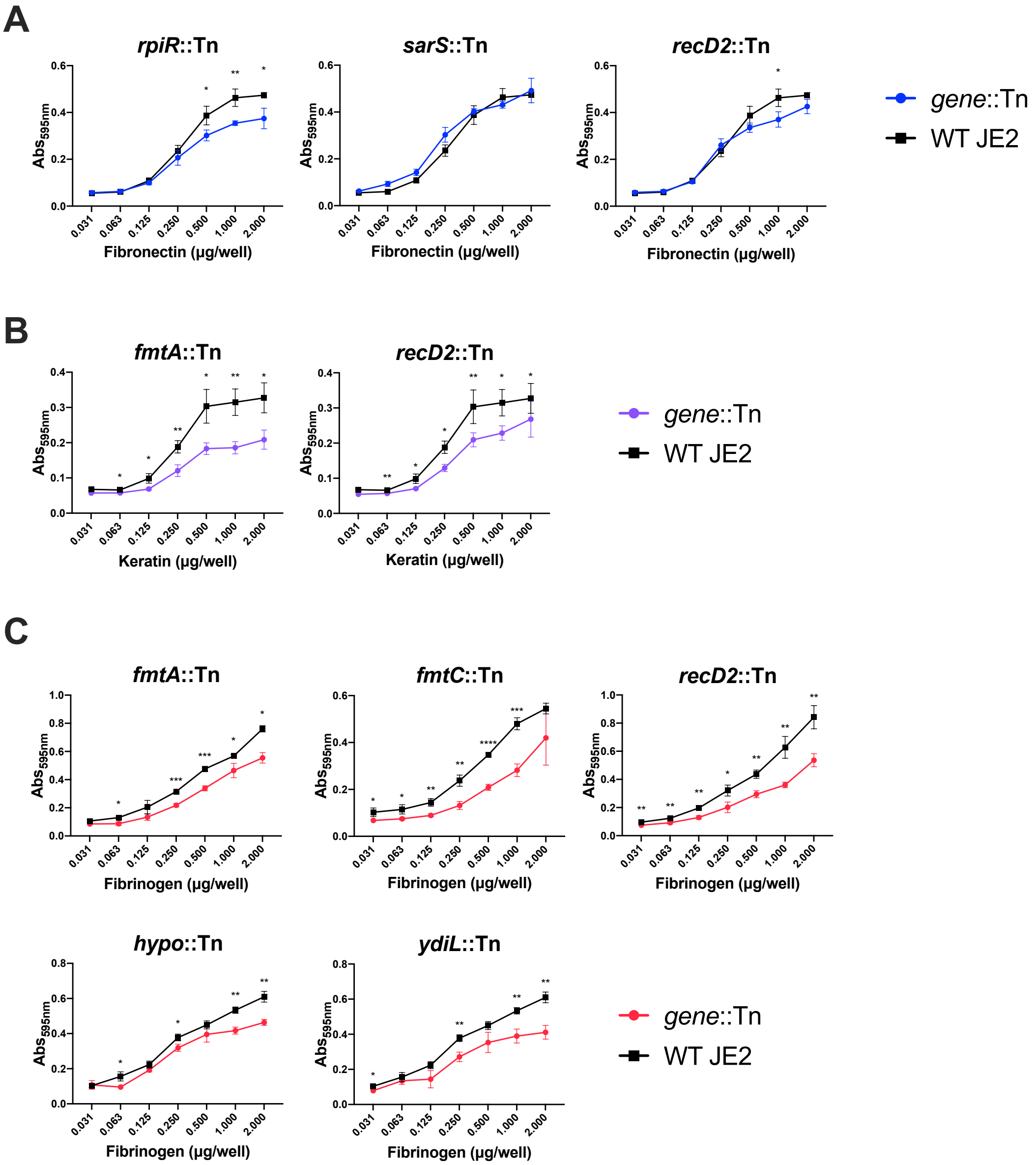

Figure S2. Crystal violet detection of poorly characterized adhesion-defective *S. aureus* mutants (A. Fibronectin, B. Keratin, and C. Fibrinogen). To confirm the adhesion defects were not due to decreased affinity of the ELISA antibodies, we profiled adhesion of the poorly characterized *S. aureus* mutants to their respective ligand(s) using crystal violet as an alternative detection method. Each ligand was titrated (A-C), the strains (shown in each individual graph) were propagated in 10 mL TSB to the appropriate OD_{600nm}, with three biological replicates. Crystal violet was used to assess adhesion (Abs_{595nm}) and the values shown are the average of three biological replicates ± the standard deviation. *P*-values were calculated using the two-tailed unpaired Student's *t*-test. Statistically significant decreases, compared to wild-type JE2, were denoted as $P \leq 0.05$*; $P \leq 0.01$**; $P \leq 0.001$***; $P \leq 0.0001$****.
